# Detection of Human Microchimerism following Allogeneic Cell Transplantation Using Droplet Digital PCR

**DOI:** 10.1155/2019/8129797

**Published:** 2019-06-12

**Authors:** Catherine A. Lombard, Alexandre Fabre, Jérôme Ambroise, Joachim Ravau, Floriane André, Nawal Jazouli, Mustapha Najimi, Xavier Stéphenne, Françoise Smets, Jean-Luc Vaerman, Etienne M. Sokal

**Affiliations:** ^1^Laboratory of Pediatric Hepatology and Cell Therapy, Institut de Recherche Expérimentale et Clinique, Université Catholique de Louvain, 1200 Brussels, Belgium; ^2^Service de Pédiatrie Multidisciplinaire, Hôpital de la Timone, APHM, Marseille, France; ^3^Aix Marseille University, INSERM, MMG, Marseille, France; ^4^Centre de Technologies Moléculaires Appliquées (CTMA), Institut de Recherche Expérimentale et Clinique, Université Catholique de Louvain, 1200 Brussels, Belgium; ^5^Service de Gastroenterologie & Hépatologie Pédiatrique, Cliniques Universitaires St Luc, 1200 Brussels, Belgium; ^6^Département de Biologie Clinique, Cliniques Universitaires St Luc, 1200 Brussels, Belgium

## Abstract

**Background:**

Cell transplantation is in clinical development for the treatment of various ailments including acquired and inborn hepatic diseases. Detection and quantification of the donor cells after infusion remain difficult. Traditional methods (sex-based FISH, HLA mismatch, and Short Tandem Repeat PCR) can only achieve low levels of sensitivity (1%) and therefore are seldom used. The use of a droplet digital PCR (ddPCR) assay based on mismatch of null alleles is a promising alternative.

**Methods:**

We selected genes with a high frequency of null genotype in the general population (SRY, RHD, TRY6, LEC3C, GSTM1, and GSTT1) and investigated their expression by liver progenitor cell donors and liver cell therapy recipients, in order to identify genes of interest for each donor/recipient couple. We first validated the detection of microchimerism by ddPCR and then used these assays to detect and quantify microchimerism in pre- and postinfusion liver biopsies.

**Results:**

We validated the ddPCR detection of the selected genes based on linearity, precision, lack of inhibition, and accuracy, and we established limits of blank, limits of detection, and limits of quantification to ensure the reliability of the results. After genotyping donors and recipients, we were able to identify at least one gene of interest for each donor/recipient couple. We detected donor cells in the three patients posttransplantation. However, analysis of several biopsies taken at the same timepoint revealed a heterogeneous cell distribution. In addition, the values obtained remained below the limit of quantification. Therefore, the actual quantification of microchimerism may not be entirely accurate.

**Conclusions:**

Overall, our study demonstrates that the detection of microchimerism post-liver cell transplantation can be performed using ddPCR amplification of null allele genes expressed by the donor but absent from the recipient. However, this technique can be extended to other cell types and target organs in cell transplantation.

## 1. Introduction

Cell transplantation is in clinical development for the treatment of various ailments including inborn and acquired liver diseases. In this context, the detection and quantification of the donor cells in the recipient liver (microchimerism) are essential steps to be able to correlate the presence/absence and magnitude of a clinical effect with the presence/absence and number of donor cells. However, the number of cells injected represents a small percentage of the total recipient organ's mass [1]. In addition, regardless of their origins and target organs, mesenchymal stem cells (MSCs) have shown a fairly low level of engraftment so far [2–6]. Finally, available tissue samples are usually restricted to a small biopsy taken at random. Therefore, a reliable technique with high specificity and low limit of detection is needed.

Several techniques have been tested by our laboratory and others. Fluorescence in situ hybridization (FISH) relies on fluorescent probes to detect specific DNA or RNA sequences. We have previously used this method to demonstrate the presence of the Y chromosome on a liver-biopsy fingerprint when a female recipient was infused with male donor cells [7]. However, the fingerprint only gives access to a limited number of cells (about 200), which limits sensitivity (the lowest number of cells that could theoretically be detected is 1 in 200, corresponding to 0.5%). In addition, Y chromosome-based FISH can only be used in case of male donor and female recipient, which limits its application. PCR-based methods are also available to detect not only a sex mismatch but also a HLA mismatch, Short Tandem Repeats (STR), Single Nucleotide Polymorphisms (SNPs), or insertion-deletion (Indel) markers [8–11]. The STR approach only allows for the detection of up to 1-5% of chimerism, but real time-PCR using either SNPs or Indels can achieve 0.1-0.01% sensitivity [8, 9]. The use of null alleles (total absence of the gene) is particularly interesting because of the theoretical absence of nonspecific signal. Null alleles include not only SRY and RHD but also genes such as TRY6, LEC3C, GSTM1, or GSTT1 [9, 12, 13]. However, the disadvantages of real-time PCR are the loss of linearity at small concentrations, which are of interest in the detection of chimerism (our own observations), and the necessity to establish a cut-off value to eliminate background noise, which may lead to a loss of information.

Droplet digital PCR (ddPCR) uses a water-oil emulsion principle to partition the samples into 20000 droplets of theoretically equal volume, which will each contain from 0 to a few copies of the template DNA and behave as an individual PCR reaction. The amount of template DNA in the sample can then be derived from the number of positive (fluorescent) droplets using Poisson's statistics without the need for a standard curve [14–16]. The technique also offers the advantage of being less susceptible to inhibition than real-time PCR and more sensitive for the detection of low amounts of DNA [14, 15].

Droplet digital PCR has already been used in a variety of applications such as detection of microchimerism based on Indels or STRs in blood samples and quantification of circulating donor DNA in solid transplant recipients [17–20]. In addition, it was recently described for the detection of donor/recipient chimerism in the blood following hematopoietic stem cell transplantation [21].

Therefore, we decided to apply the technique to the detection of microchimerism in liver biopsies of patients transplanted with allogeneic adult-derived liver cells, using null alleles. In this paper, we describe the validation of the technique and its application to clinical samples from LCT patients.

## 2. Materials and Methods

Three patients received ADHLSC-based LCT under hospital exemption rules. ADHLSC-based LCT was approved by the Ethics Committee of Cliniques Universitaires Saint-Luc and informed consent/assent for cell transplantation was obtained from the parents and/or the patient.

### 2.1. LCT and Follow-Up

#### 2.1.1. Patient 1

A 17-year-old patient, who weighted 63.9 kg, received seven infusions of ADHLSCs for a total of 3 billion cells over 3 days (mean viability of cells after thawing: 88.2%) representing about 0.8% of the total of liver cells (more details in [22]) [23]. The patient was maintained under tacrolimus (Prograf, Astellas, Belgium) to reach a trough level of 6-8 ng/ml and received 1 mg/kg/day of prednisolone during the infusions. Needle biopsies of the liver were taken before LCT and at 3 months (right and left) and 7.5 months post LCT. The samples were stored frozen at -80°C.

#### 2.1.2. Patient 2

A 22-year-old patient, who weighed 79.5 kg, received five infusions of ADHLSCs over three days for a total of 2.2 billion cells, with an average viability of 83%. The immunosuppressive regimen comprised tacrolimus to reach a trough level of 6-8 ng/ml and 1 mg/kg prednisolone (Pfizer, Belgium) on the day of infusion, which was decreased to 0.2 mg/kg Medrol postinfusion. Needle biopsies of the liver were taken before ADHLSC infusion and at 3 months postinfusion. The patient received a liver transplant 5 months post ADHLSC-based LCT, and the liver was recovered and cryopreserved as slices taken from the left to the right lobe. The samples were stored frozen at -80°C.

#### 2.1.3. Patient 3

A 3-year-old girl who had previously received hepatocyte-based LCT (see [2] for details) was then treated with ADHLSC-based LCT.

At the time of the ADHLSC infusions, the child was weighing 14.9 kg. She first received two infusions of 262 and 230 million cryopreserved/thawed ADHLSCs and received a third infusion of 430 million fresh ADHLSCs two weeks later. The immunosuppression regimen based on tacrolimus remained identical to reach trough levels 6-8 ng/ml [2]. Needle biopsies of the liver were taken before ADHLSC infusion and at 3.5 months postinfusion. The child received a liver transplant 10 months post ADHLSC-based LCT, and the liver was recovered and cryopreserved as slices taken from the left to the right lobe. The samples were stored frozen at -80°C.

### 2.2. ADHLSC Isolation, Formulation, and Infusion

ADHLSCs were obtained from the culture of thawed liver parenchymal cells isolated from healthy cadaveric donors as previously described [24]. Successive cultures were maintained until the fifth-sixth passage (P5-P6). From those passages, cells were cryopreserved at a concentration of 2-4.10^6^ cells/ml in FBS solution supplemented with 10% DMSO. The cell suspensions were cryopreserved using a programmed freezer equipment (Cryoson) using a stepwise decrease in temperature (1°C/min until -40°C and then 2°C/min until -90°C). Upon reaching -90°C, the cells were transferred to liquid nitrogen tanks. For infusions, cells were thawed at 37°C for a few minutes, washed twice with human albumin 5% solution (Hibumine, Baxter) supplemented with 5G/100 ml glucose (Sterop, Bruxelles, Belgium), 84 mg/ml sodium bicarbonate (Natribic, Sterop), 10 UI/ml heparin (Heparin Leo, Leo Pharma, Lier, Belgium), and 10 mM N-acetylcysteine (Lysomucyl, Zambon, Jette, Belgium), and resuspended to reach a concentration of 5-10.10^6^ cells/ml. The cells were then administered through a percutaneous intraportal catheter.

### 2.3. Genomic DNA Extraction

Genomic DNA (gDNA) was extracted from blood, cells, or tissue biopsies using the QIAamp® DNA Mini Kit (QIAGEN, Venlo, Netherlands) according to the manufacturer's instructions.

DNA concentration was assessed by fluorometry with a Qubit® system (Invitrogen, Thermo Fisher Scientific).

DNA extraction and quantification were performed in an accredited laboratory, which follows the ISO 15189 norm, using their validated procedures.

### 2.4. Genotyping by qPCR

Genes frequently deleted in the general population were selected for the study (see Table 1 for frequency of null genotype): SRY, GSTM1, GSTT1, RHD, LEC3C, and TRY6, and their expression evaluated by qPCR in both the donor cells and the LCT recipients.

To this end, FAM-labelled assays for the gene of interest were used in duplex with the HEX-labelled assay for the reference gene RNAse P (Integrated DNA Technologies (IDT), Leuven, Belgium). Sequences of the primers and probes used can be found in Table 2.

PCR was performed in a total volume of 20 *μ*l containing TaqMan® Gene Expression Master Mix (Applied Biosystems, Thermo Fisher Scientific), primers and probes for the gene of interest and the reference gene, and 30-120 ng of genomic DNA and Tris-EDTA (TE) buffer (IDT).

The thermocycling was performed with adequate controls on a LightCycler® 480 System (Roche Diagnostics, Basel, Switzerland) with the following protocol: incubation for 2 min at 50°C, followed by denaturation for 10 min at 95°C and 40 amplification cycles of 15 s at 95°C and 1 min at 60°C (acquisition). gBlocks gene fragments were used as positive controls (IDT). An ultrapure salmon sperm DNA solution (Invitrogen, Thermo Fisher Scientific) was used as a negative control, as well as “no template controls,” wherein the DNA was replaced by TE buffer.

### 2.5. Detection of Human Chimerism by ddPCR

A duplex digital PCR was performed using the QX200™ Droplet Digital™ PCR System (Bio-Rad Laboratories, Temse, Belgium) to measure the level of donor/recipient chimerism in the liver biopsies.

SRY, RHD, GSTM1, GSTT1, LEC3C, and TRY6 assays were used to quantify copies of DNA in samples of interest. The RNAse P gene was used as a reference gene.

The ddPCR reaction mixture consisted of ddPCR™ Supermix for probes, no dUTP (Bio-Rad Laboratories), assays for the gene of interest and the reference gene, 30-120 ng of total DNA and TE buffer. For each reaction, 20 *μ*l of reaction mixture were loaded onto a disposable plastic cartridge (Bio-Rad Laboratories) with 70 *μ*l of droplet generation oil (Bio-Rad Laboratories), covered with a specific gasket (Bio-Rad Laboratories), and placed in the droplet generator (Bio-Rad Laboratories). gBlocks were used as positive controls. An ultrapure salmon sperm DNA solution was used as a negative control, as well as “no template controls,” where the DNA was replaced by TE buffer.

Droplets generated from each sample were transferred to a semiskirted 96-well twin.tec PCR plate (Eppendorf, Hamburg, Germany), and PCR amplification was performed on a T100™ Thermal Cycler (Bio-Rad laboratories) with the following protocol: 95°C for 10 min, then 40 cycles of denaturation at 94°C for 30 s and annealing/extension at 60°C for 1 min (temperature ramp rate of 2.5°C/sec), followed by enzyme deactivation at 98°C for 10 min and storage at 4°C.

After amplification, the plate was loaded onto the QX200™ Droplet Reader (Bio-Rad Laboratories), and the droplets from each well were automatically read. Data were analyzed with the QuantaSoft™ analysis software (Bio-Rad Laboratories). A threshold was set based on the resolution of positive and negative droplets so as to eliminate background noise (representative data are shown in Supplementary Figure S1). The quantitation of target molecules was presented as a ratio of the number of droplets positive for the gene of interest over the number of droplets positive for the reference gene RNAse P (Supplementary Figure S1D). The ratio indicates the quantity of infused cells in a patient biopsy post infusion. Indeed, a ratio of 1.4*E*-04 means the detection of 14 infused cells for 100,000 cells in a biopsy, corresponding to a donor/recipient chimerism of 0.014%.

### 2.6. Validation of the Chimerism Detection Method by ddPCR

In prevalidation steps, annealing temperatures from 56°C to 66°C were tested to choose the best condition to increase the detection (signal to noise ratio − amplitude of the droplets) (data not shown). The annealing temperature of 60°C, giving the best amplitude/signal, was chosen.

The following criteria were then analyzed for validation of the method: inhibitions, linearity, precision, specificity, accuracy, limit of blank (LoB), limit of detection (LoD), and limit of quantification (LoQ), as well as interassay and interoperator precision. The methods concerning the evaluation of inhibitions, precision, and accuracy can be found in supplementary data.

#### 2.6.1. Artificial Chimerism Samples

Artificial chimerism samples were generated by mixing a known amount of gDNA from a positive PBMC sample with a negative PBMC gDNA sample and performing a serial dilution.

#### 2.6.2. Linearity

The linearity of the method was evaluated using decreasing percentages of artificial chimerism (120 ng of DNA) and was considered acceptable if the regression coefficient was >0.95.

#### 2.6.3. Specificity

The specificity of a qPCR assay is defined as the ability to generate one and only one amplicon product that is the intended target sequence. The specificity of the TaqMan® assays used was first evaluated by running the qPCR products on agarose gels to visualize them, followed by sequencing. Second, we evaluated the ability to distinguish true positives from false positives resulting from background noise generated by low DNA samples. To this end, 8 negative samples (ultrapure salmon sperm DNA), 8 NTC samples, and 2 positive samples were analyzed by ddPCR in 2 to 3 separate runs using 120 ng of DNA.

#### 2.6.4. Limit of Blank, Limit of Detection, and Limit of Quantification

To evaluate these parameters, three identical plates were run with 8 replicates each of a serial dilution of artificial chimerism samples (120 ng of DNA containing 100%, 25%, 6.25%, 1.56%, 0.39%, 0.098%, 0.024%, 0.0061%, and 0% of input donor DNA) for each gene of interest.

*(1) Limit of Blank*. The limit of blank (LoB) is defined as the highest apparent value (ratio between the number of droplets obtained for the gene of interest and the number of droplets obtained for the reference gene RNAse P) expected from a blank sample containing no analyte [25]. A result above or equal to the LoB is to be considered as a positive result, while a result below the LoB is to be considered as negative. In this study, the blank samples were DNA samples with null genotype for the gene of interest. The LoB was calculated based on the results of the 24 replicates of the blank using the formula LoB = mean blank + 1.645 standard deviation (SD) from the blank, which warranties a 95% specificity, which means that 95% of negative samples will be below the LoB and recognized as negative samples. The LoB is expressed as a ratio.

*(2) Limit of Detection*. The limit of detection (LoD) is defined as the lowest analyte concentration (chimerism percentage) likely to be reliably distinguished (i.e., with statistical strength) from the LoB and at which a detection is feasible [25]. The LoD is determined by utilizing both the measured LoB and test replicates of a sample known to contain a low concentration of analyte using the formula LoD = LoB + 1.645 (SD low concentration sample). The LoD gives information on the percentage of chimerism required to ensure that most (95%) of the positive samples truly be considered positive. The objective was to reach a LoD < 0.024%.

*(3) Limit of Quantification*. The limit of quantification (LoQ) is defined as the concentration at which detection can be reliably performed and at which predefined parameters to prevent imprecision and bias are met [25]. In our case, the LoQ corresponds to the percentage of chimerism for which all the positive samples give a positive signal and the CV remains below 20% in terms of copy numbers.

## 3. Results

### 3.1. Genotyping and Identification of Genes of Interest for the Detection of Donor/Recipient Microchimerism

In order to detect donor cells in the recipient's liver, we needed to identify the gene(s) expressed by the donor but absent in the recipient (informative genes) so that the presence of the gene(s) in the recipient posttransplantation could be correlated with the presence of the donor cells.

Donors and recipients were genotyped by qPCR and assigned one of the following statuses: homozygous positive (+/+), heterozygous positive (+/-), or null (-/-) (Table 3). Donors were considered positive for the gene regardless of the homozygous or heterozygous status.

Patient 1 had a null genotype for GSTM1 and GSTT1 but was positive for SRY, TRY6, RHD, and LEC3C. The ADHLSC donor (infused cells) was heterozygous for GSTM1 and homozygous positive for GSTT1, which were therefore chosen as informative genes.

Patient 2 had a null genotype for TRY6 and LEC3C and was positive for all the other genes. The donor was null for LEC3C but homozygous positive for TRY6. TRY6 was therefore identified as the informative gene for this patient.

Patient 3 had a null genotype for SRY, RHD, GSTM1, and LEC3C and was positive for GSTT1 and TRY6. The ADHLSC donor was heterozygous for SRY and homozygous positive for RHD and GSTM1. Therefore, SRY, RHD, and TRY6 were chosen as informative genes.

### 3.2. Evaluation of Donor/Recipient Microchimerism by ddPCR

#### 3.2.1. Validation of the Technique

**Figure 1:**
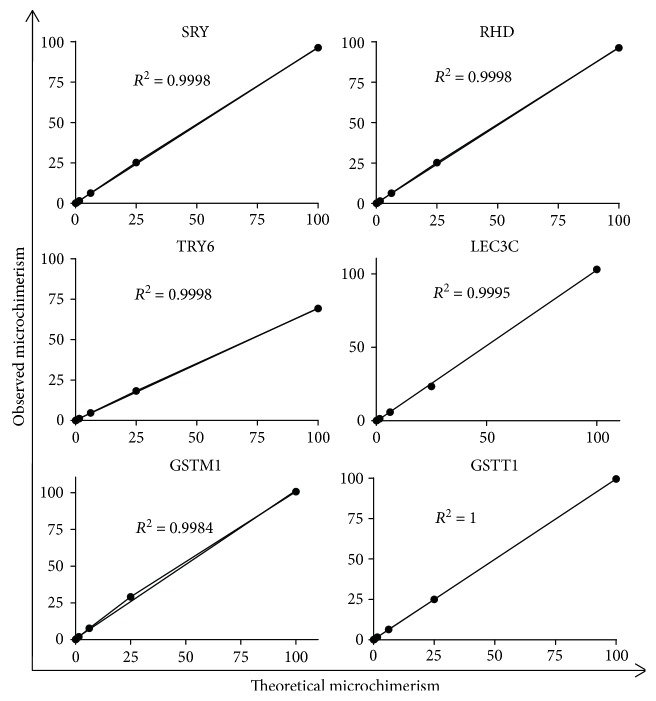
Demonstration of ddPCR linearity for the various genes tested. Donor DNA was serially diluted and used in a ddPCR assay for SRY, RHD, TRY6, LEC3C, GSTM1, and GSTT1. The percentages of chimerism obtained (observed microchimerism) were plotted against the expected values (theoretical microchimerism) and the regression coefficient (*R* ^2^) calculated.

The linearity of the ddPCR assays was evaluated by analyzing 8 replicates of the 8 concentrations of artificial microchimerism in 3 independent experiments. The mean observed microchimerism values were then plotted against the theoretical microchimerism. For SRY, which can only have a maximum value of 50% (for a man), the values obtained were multiplied by 2. As expected, the linearity of the assays was extremely good (*R* ^2^: SRY 0.9998, RHD 0.9998, TRY6 0.9998, LEC3C 0.9995, GSTM1 0.9984, and GSTT1 1) (Figure 1 and Table 4).

The specificity tests ran in the prevalidation step showed that the qPCR assays all yielded a single product, which had the expected sequence (data not shown). For the validation of the ddPCR, 8 replicates of ultrapure salmon sperm DNA and NTCs (blank sample) were also analyzed in 3 independent experiments to study the false-positive rate. The false-positive rate was null only for the detection of SRY and RHD (Table 5). One to two positive droplets out of more than one million generated droplets were found in a total of 24 wells for the other genes (TRY6: 1, LEC3C: 1, GSTM1: 2, and GSTT1: 2). In addition, a few droplets were also positive for RNAse P in the blank samples and the no template controls. However, the abundance of positive droplets was always at least 500 times greater in the positive samples than in the negative and blank samples, allowing us to distinguish true positives from background noise.

Nonetheless, the fact that the false-positive rate was not null for all assays led us to calculate a limit of blank (LoB) above which the signal detected can be considered a true positive and which would be used to calculate a limit of detection (LoD) and a limit of quantification (LoQ) for each assay. In total, 24 replicates of the 8 concentrations of artificial microchimerism and three homozygous negative samples were used to precisely generate LoB, LoD, and LoQ parameters for each gene of interest (Table 6). The LoDs obtained were below 0.024%, meaning that we should be able to detect the donor cells as long as the amount injected or present at the time of the test was above 0.024% of the total liver of the recipient.

#### 3.2.2. Human Microchimerism Detection

For patient 1, two informative genes were used. As expected, there was no signal for GSTM1 or GSTT1 in the preinfusion biopsy (Table 7(a)). At 3 months, a positive signal was detected for both GSTM1 and GSTT1. The microchimerism was evaluated at 0.02596% for GSTT1 and 0.03308% for GSTM1, respectively. However, it has to be noted that the values obtained remained below the respective LoQs. There was no signal detected in the biopsy taken at 7.5 months postinfusion.

For patient 2, only one informative gene could be used. As expected, there was no signal for TRY6 in the preinfusion biopsy (Table 7(b)). At 3 months, a signal was detected but it fell under the LoB. The sample was, therefore, considered negative for donor cells. At 5 months, a signal over the LoB was detected and the microchimerism was evaluated at 0.01532% in one of the serial liver slices. However, this value was below the LoQ. In addition, the signals detected in the other 7 slices were all under the LoB and the results were consequently considered as negative for these samples.

For patient 3, three informative genes were used. No biopsy sample taken before hepatocyte infusion was available for analysis of that baseline. There was no signal for SRY or GSTM1 in the biopsy taken before ADHLSC infusion (but after hepatocyte infusion) (Table 7(c)). However, a signal above the LoB was found for RHD, indicating cell detection. As no ADHLSC had been injected yet, we could only assume that the DNA detected resulted from the hepatocytes previously injected. Indeed, both hepatocyte donors were homozygous for that gene. Looking at the results in the seven 10-month post-ADHLSC infusion biopsies, we detected human cells (signal > LoB) with at least one of the three genes in 2 out of 7 samples. In fact, one biopsy was positive for RHD alone and the other for RHD in combination with SRY. However, the values obtained were below the LoQs. No signal was detected for GSTM1.

## 4. Discussion

The detection and quantification of microchimerism following allogeneic cell infusion remain a hurdle in cell therapy. In this paper, we describe a robust and specific method based on the analysis of null allele expression by droplet digital PCR.

DNA extraction and quantification were performed in an accredited laboratory, which follows the ISO 15189 norm, using their validated procedures, so as to ensure the highest quality work. In addition, the genotyping by qPCR and the detection of chimerism by ddPCR were performed using our procedures in the accredited laboratory. We first validated the genotyping procedure (data not shown) and then genotyped the donors and recipients of the study. In this study, donors were considered positive for the gene regardless of the homozygous or heterozygous status. Indeed, even if a heterozygous status means a lower capacity to detect the microchimerism because of smaller quantities of DNA per cell, the results of ddPCR can be corrected for the heterozygous genotypic status by multiplying the results obtained with heterozygous genes by two.

Various parameters were evaluated to validate the ddPCR technique. We found an excellent linearity, as demonstrated by the coefficients of determination obtained. The technique also showed good precision, with intraoperator CV below 10% and interoperator CV below 12% for all genes tested. In addition, the assays were considered free of inhibition, as the variations between the tests ran with 120 ng and 12 ng of input DNA remained below 10% (except for one biopsy tested for TRY6 which showed a variation of 11.6%). Despite detecting a few positive droplets in the blank or no template controls for some of the genes of interest, the abundance of positive droplets in the positive controls was always such that we were able to clearly distinguish true positives from background noise. Therefore, the specificity of the technique was considered adequate. Overall, the technique also showed good accuracy in the small percentages of microchimerism that we can expect to see in the patients, with a difference between theoretical and observed values below 20% for RHD, LEC3C, GSTM1, and TRY6 and below 23% for GSTT1. SRY showed the most inaccuracy, with differences above 20%. However, according to the firm's technical note, the fluorimetric method used to measure the DNA concentration (Qubit), while more accurate than techniques based on UV absorbance such as Nanodrop, can show a deviation from the actual concentration of up to 20%, which could partially explain the relative inaccuracy.

In their paper, Okano et al. stated that the analysis of SRY in ddPCR gave good accuracy [21]. However, the approach used to validate the accuracy was different from ours. First, they correlated the results of clinical samples obtained with ddPCR to those obtained with other analytical methods such as FISH and STR-PCR. Second, mixes of male and female DNA were used to generate a correlation coefficient between observed and theoretical values of chimerism. They did not give the actual values obtained or calculate the %CV between observed and theoretical values. However, the correlation coefficient could be good even if the accuracy is not satisfactory. Indeed, our *R* ^2^ for SRY was 0.9998, and yet, the differences between theoretical and observed values were sometimes quite large. In addition, the lowest percentage of chimerism that they tested was 0.78, which is much higher than the lowest point of our artificial chimerism curve.

Patient 1, weighing 63 kg, received 3 billion cells; patient 2, weighing 80 kg, received 2.2 billion cells; while patient 3, weighing 15 kg, received 922 million cells. If we consider that the liver of a 70 kg adult contains 361 billion cells, the patients received between 0.5 and 1.2% of the total cell number in the liver, which is higher than the limits of detection that were calculated for the different genes [23]. Therefore, in theory, the patients have received enough cells to enable us to detect them.

The LoB established allowed to safely determine positive samples. However, the percentages of microchimerism obtained when detection was possible were all below the LoQ for the corresponding gene. Therefore, the quantification cannot be considered entirely reliable, and the microchimerism of some of the samples may have been underestimated.

Analysis of multiple biopsies from the same donor allowed us to show that the distribution of the cells throughout the liver is not homogeneous. Indeed, for the same patient, some samples appeared negative, while others, taken at the same time point postinfusion, showed positive results.

We analyzed all the genes of interest available for each patient, based on their genotype and that of the donor cells, and investigated whether the results were consistent. Surprisingly, we found that the results obtained with one gene did not always correlate with the results obtained with another in the same biopsy. It seems counterintuitive that a same biopsy analyzed using two or three different genes would give a negative result with one and a positive result with the other. However, one has to consider that each gene is analyzed in a different ddPCR well, which can be seen as a subfraction of the overall sample. It is therefore possible that one well would contain one or a few copies of donor DNA while the other would not contain any. The results would probably better correlate if all the genes could be analyzed in the same well using a multiplex assay.

Overall, our study also highlights the importance of selecting a donor based not only on the cells' potency but also on their genotype, if one wants to be able to detect and quantify the injected cells. It would be tempting to suggest favoring the injection of male cells into female recipients, as these genotypes can be assessed noninvasively. However, in our experience, SRY was the assay with the poorest accuracy, even at percentages of chimerism above the LoQ. RHD, on the other hand, showed really good accuracy at all the percentages of microchimerism tested and its genotype can also be assessed with a simple and minimally invasive test already routinely performed by hospitals.

One of the pitfalls of the technique, as of all PCR-based techniques, is that it can only measure amounts of donor DNA but cannot give any information on whether the cells are alive or dead. In the absence of clinical effect, it is therefore impossible to tell if the cells are present but have lost functionality or if the DNA detected comes from dead cells.

In patient 1, we saw a drastic decrease in chimerism between 3 months and 7 months postinfusion. It could be due to the heterogeneous distribution of the cells throughout the liver, but it also could be related to an episode of acute enteritis with diarrhea, which developed five months after infusion, leading to the activation of the immune system and the potential clearing of the cells. Our previous studies suggest that ADHLSCs are not immunogenic in vitro [26–28]. However, little is known about their immunogenicity *in vivo*, especially in the context of an ongoing inflammatory response. Indeed, experiments subjecting liver progenitor cells to inflammatory conditions *in vitro* triggered an upregulation of both costimulatory molecules such as CD40 and immunosuppressive molecules such as CD274 [28, 29].

Patient 3 had received hepatocyte infusions before she received ADHLSC infusions. Unfortunately, the genotype of the hepatocytes and that of the ADHLSCs received overlapped for RHD expression. Therefore, it is impossible to determine if the RHD signal detected comes from the hepatocytes or the ADHLSCs. It is nonetheless noteworthy that there was a signal for RHD in the biopsy taken between the hepatocyte and ADHLSC infusions, showing the presence of DNA from the donor hepatocytes several months after the infusions. Unfortunately, this result did not correlate with clinical improvement at that time, underlying the potential detection of dead cells.

## 5. Conclusion

In conclusion, we have demonstrated that the detection of microchimerism following cell infusion can be achieved successfully with the use of a procedure based on ddPCR analysis of null alleles. The technique can be used for LCT but also extended to other types of cell transplantation and target organs. Development of multiplex analyses would likely strengthen the results.

## Figures and Tables

**Table 1:** Frequency of null phenotype for the six genes of interest.

Gene	Full name	Homozygous null (%)	Reference
RHD	Rh blood group, D antigen	10	[9]
TRY6 (PRSS3P2)	Protease, serine 3, pseudogene 2	17^∗^	[12]
GSTM1	Glutathione S-transferase mu 1	59^∗^	[12]
GSTT1	Glutathione S-transferase theta 1	15^∗^	[12]
SRY	Sex-determining region Y	50	[9]
LCE3C	Late cornified envelope 3C	31^∗^	[12]

^∗^Calculated using the Hardy-Weinberg equilibrium for CEU population.

**Table 2:** Primers and probes.

	Probe	Primer 1	Primer 2
SRY	5′-/56-FAM/CACTTACCG/ZEN/CCCATCAACGCAG/3IABkFQ/-3′	5′-GTTACCCGATTGTCCTACAGC-3′	5′-CGAAAGCCACACACTCAAGA-3′
RHD	5′-/56-FAM/CAAAGTCTC/ZEN/CAATGTTCGCGCAGG/3IABkFQ/-3′	5′-TCAAAGAGTGGCAGAGAAAGG-3′	5′-AACTTCCTCTCACTGTTGCC-3′
TRY6	5′-/56-FAM/AGGCGCACT/ZEN/CTACCACCATGAATC/3IABkFQ/-3′	5′-CACAAAGGCAAGGATCAGGA-3′	5′-GATCCTCCAGAGCTATAAAGACG-3′
LEC3C	5′-/56-FAM/TTCTGAAAG/ZEN/TGGCTGCTGCCTGA/3IABkFQ/-3′	5′-GGCATTGATGGGACCTGAA-3′	5′-CCTCTTCTGACTGTGCTCTAAG-3′
GSTM1	5′-/56-FAM/CGGTTTAGG/ZEN/CCTGTCTGCGGAATC/3IABkFQ/-3′	5′-TATCATGGGCATGGTGCTG-3′	5′-CCCTCTCCGGAGCTCTTATA-3′
GSTT1	5′-/56-FAM/CACCATCCC/ZEN/CACCCTGTCTTCC/3IABkFQ/-3′	5′-CCTCAGTGTGCATCATTCTCA	5′-AAGTCCCAGAGCACCTCA-3′

**Table 3:** Genotypes and genes of interest.

	SRY	RHD	TRY6	LEC3C	GSTM1	GSTT1
Patient 1	HETERO+	HOMO+	HETERO+	HOMO+	*Null*	*Null*
Donor ADHLSCs 1	Null	HETERO+	HETERO+	HETERO+	*HETERO+*	*HOMO+*
Gene(s) of interest					*x*	*x*

Patient 2	HETERO+	HOMO+	*Null*	Null	HOMO+	HETERO+
Donor ADHLSCs 2	HETERO+	HOMO+	*HOMO+*	Null	HOMO+	HETERO+
Gene(s) of interest			*x*			

Patient 3	*Null*	*Null*	HETERO+	Null	*Null*	HOMO+
Donor ADHLSCs 2	*HETERO+*	*HOMO+*	HOMO+	Null	*HOMO+*	HETERO+
Gene(s) of interest	*x*	*x*			*x*	
Donor hepatocytes 1	*Null*	*HOMO+*	HETERO+		*Null*	HOMO+
Donor hepatocytes 2	*HETERO+*	*HOMO+*	HETERO+		*HETERO+*	HOMO+

ADHLSC: adult-derived human liver stem/progenitor cells; HETERO+: heterozygous positive; HOMO+: homozygous positive.

**Table 4:** Linearity.

Theoretical microchimerism (%)	Observed microchimerism (%)					
	SRY^∗^	RHD	TRY6	LEC3C	GSTM1	GSTT1
100	102.47	96.25	69.2667	103.00885	100.67	99.5
25	27.93	25.31	18.3	23.4639498	29.1	25.07
6.25	7.407	6.355	4.6767	5.88328076	7.79	6.493
1.563	1.919	1.576	1.1967	1.46233649	1.967	1.667
0.3906	0.4907	0.3854	0.3117	0.38444084	0.4877	0.413
0.0977	0.1211	0.1022	0.0793	0.09398998	0.131	0.1063
0.0244	0.0347	0.0203	0.0236	0.02184339	0.0283	0.0294
0.0061	0.0048	0.006	0.0061	0.00555927	0.0058	0.0048

^∗^Results were multiplied by 2.

**Table 5:** Specificity.

Gene	Positive droplets	Control (-)	NTC	Control (+)
SRY	Average number of SRY+ droplets	0	0	53295
	Average number of RNAse P+ droplets	0.875	0.5	49548
RHD	Average number of RHD+ droplets	0	0	10140
	Average number of RNAse P+ droplets	0	0.0833	44913
TRY6	Average number of TRY6+ droplets	0.0417	0	7017
	Average number of RNAse P+ droplets	0.25	0.125	39515
LEC3C	Average number of LEC3C+ droplets	0.0417	0.0417	1325.666667
	Average number of RNAse P+ droplets	0.5833	0.3333	6477
GSTM1	Average number of GSTM1+ droplets	0.0417	0.0417	11861.66667
	Average number of RNAse P+ droplets	20.75	0.4167	11417.75
GSTT1	Average number of GSTT1+ droplets	0.0833	0	12025.5
	Average number of RNAse P+ droplets	0.9583	0.0417	12123.75

NTC: no template control.

**Table 6:** LoB-LoD-LoQ.

Gene	LoB (ratio)	LoD	LoQ
SRY	0.0000276	0.01800%	0.39000%
RHD	0.0000171	0.01290%	0.39000%
TRY6	0.0000562	0.01650%	0.39000%
LEC3C	0.0000263	0.01000%	0.09800%
GSTM1	0.000033	0.01330%	0.09800%
GSTT1	0.000035	0.01230%	0.39000%

LoB: limit of blank; LoD: limit of detection; LoQ: limit of quantification.

**Table:** (a) Patient 1

Samples	GSTM1			GSTT1		
	Ratio	>LoB?	% chimerism	Ratio	>LoB?	% chimerism
Preinfusion biopsy	0	No	NA	0	No	NA
3M postinfusion biopsy	**0.0001654**	**Yes**	**0.03308**	**0.0002596**	**Yes**	**0.02596**
7.5M postinfusion biopsy	0	No	NA	0	No	NA

**Table:** (b) Patient 2

Samples	TRY6		
	Ratio	>LoB?	% chimerism
Preinfusion biopsy	0	No	NA
3M postinfusion biopsy	0.00000857	No	NA
5M postinfusion biopsy 4	0.0000545	No	NA
5M postinfusion biopsy 5	0.0000175	No	NA
5M postinfusion biopsy 7	0.0000144	No	NA
5M postinfusion biopsy 9	0.0000339	No	NA
5M postinfusion biopsy 13	0.0000074	No	NA
5M postinfusion biopsy 16	0.00000649	No	NA
5M postinfusion biopsy 18	**0.0001532**	**Yes**	**0.01532**
5M postinfusion biopsy 19	0.0000149	No	NA

**Table:** (c) Patient 3

Samples	SRY			RHD			GSTM1		
	Ratio	>LoB?	% chimerism	Ratio	>LoB?	% chimerism	Ratio	>LoB?	% chimerism
Preinfusion biopsy^∗^	0	No	NA	0.0000237	**Yes**	NA	0	No	NA
10M postinfusion biopsy 1	**0.0000429**	**Yes**	**0.00858**	**0.0000705**	**Yes**	**0.00705**	0.0000317	No	NA
10M postinfusion biopsy 2	0.0000182	No	NA	0.0000139	No	NA	0.0000194	No	NA
10M postinfusion biopsy 3	0.0000093	No	NA	**0.0000231**	**Yes**	**0.00231**	0.0000051	No	NA
10M postinfusion biopsy 4	0.0000199	No	NA	0	No	NA	0	No	NA
10M postinfusion biopsy 5	0.0000126	No	NA	0.0000127	No	NA	0.0000085	No	NA
10M postinfusion biopsy 6	0.0000056	No	NA	0.0000102	No	NA	0.0000096	No	NA
10M postinfusion biopsy 7	0.0000252	No	NA	0.0000128	No	NA	0.0000044	No	NA

## Data Availability

The data used to support the findings of this study are available from the corresponding author upon request.

## Supplementary Materials

Supplementary Materials Supplementary Materials and Methods: validation of the chimerism detection method by ddPCR: inhibitions, precision, and accuracy. Supplementary results: evaluation of donor/recipient microchimerism by ddPCR. Validation of the technique. Supplementary Table S1: precision. Supplementary Table S2: inhibitions. Supplementary Table S3: accuracy. Supplementary Figure S1: representative images of the ddPCR results obtained with the QuantaSoft software.
