# Supplementary material for: Detection of Human Microchimerism following Allogeneic Cell Transplantation Using Droplet Digital PCR
Source: Stem Cells Int. 2019 Jun 12;2019:8129797. doi: 10.1155/2019/8129797 (PMC6594293; doi:10.1155/2019/8129797)
Supplement: Supplementary Materials — and Methods: validation of the chimerism detection method by ddPCR: inhibitions, precision, and accuracy. Supplementary results: evaluation of donor/recipient microchimerism by ddPCR. Validation of the technique. Supplementary Table S1: precision. Supplementary Table S2: inhibitions. Supplementary Table S3: accuracy. Supplementary Figure S1: representative images of the ddPCR results obtained with the QuantaSoft software. [file 8129797.f1.pdf]

SUPPLEMENTARY MATERIALS AND METHODS

Validation of the chimerism detection method by ddPCR

Inhibitions

Biopsy samples positive for the gene of interest or spiked with the corresponding gDNA were analyzed in ddPCR using 120 and 12ng of DNA. The results were expressed as the ratio of the number of droplets positive for the gene of interest over the number of droplets positive for RNAse P.

Precision

Precision is defined as the degree to which repeated measurements under unchanged conditions show the same results [1]. To test the technique's precision, we verified that the results obtained were independent of the timing of the experiment and the operator. To this end, two identical ddPCR analyses were performed by the same operator a few days apart, and two were performed by two different operators using 120ng of DNA from samples with 1.56% of input donor DNA.

Accuracy

Accuracy is defined as the degree of closeness of a measured value to the true value [1]. The accuracy of the assay was evaluated using artificial chimerism samples (120ng of DNA) corresponding to input donor DNA of 100%, 25%, 6.25%, 1.56%, 0.39%, 0.098%, 0.024% and 0.0061%.

SUPPLEMENTARY RESULTS

<u>Evaluation of donor/recipient microchimerism by ddPCR</u>

Validation of the technique

Precision of the ddPCR method was assessed by two different operators in 3 independent experiments looking at repeatability (intra-operator precision) and reproducibility (inter-operator precision) using artificial microchimerism samples containing 1.56% of input donor DNA. The CVs obtained were all below 13 % for intra-operator precision, and below 12% for inter-operator precision. (Table S1)

Assay	Gene	intra-operator CV	inter-operator CV
SRY Assay	SRY	8,21	11,59
	RNAseP	2,96	2,13
RHD assay	RHD	5,52	7,59
	RNAseP	3,8	2,65
TRY6 assay	TRY6	6,95	6,47
	RNAseP	2,08	2,3
LEC3C assay	LEC3C	12,48	11,77
	RNAseP	7,47	6,09
GSTM1 assay	GSTM1	4,84	7,77
	RNAseP	2,14	5,05
GSTT1 assay	GSTT1	6,06	5,71
	RNAseP	4,44	4,02

Supplementary Table S1. Precision

CV :coefficient of variation

The presence or absence of inhibitions was investigated by testing 3 different biopsies with 120ng and 12ng of DNA input and evaluating the variation in the ratio (between the number

of droplets positive for the gene of interest over the number of droplets positive for the reference gene RNAse P). We did not observe any inhibition for SRY, RHD, LEC3C, GSTM1, and GSTT1 (Table S2). One biopsy tested for TRY6 showed a slightly higher variation but it remained close to 10% (11.2%) while the other two biopsies showed acceptable variation.

Gene	Sample	Difference between 120ng and 12 ng (%)
SRY	S1	1,1
	S2	0,8
	S3	0,3
RHD	S1	-2,7
	S2	-3,8
	S3	0,2
TRY6	S1	11,2
	S2	2,7
	S3	8
LEC3C	S1	-3,2
	S2	3,6
	S3	1
GSTM1	S1	4,3
	S2	0,2
	S3	1,3
GSTT1	S1	2,7
	S2	2,7
	S3	0,8

Supplementary Table S2. Inhibitions

S : sample

The technique's accuracy was evaluated by determining the difference between the observed concentrations and the theoretical concentrations of artificial chimerism (Table S3).

	Expected percentage	observed percentage	difference	% difference (absolute value)
**SRY**	100	102,466667	2,466667	**2,47**

	25	27,933333	2,933333	**11,73**
	6,25	7,406667	1,156667	18,51
	1,52	1,918667	0,398667	26,23
	0,39	0,490667	0,100667	25,81
	0,098	0,121133	0,023133	23,61
	0,024	0,034733	0,010733	44,72
	0,0061	0,004780	-0,001320	21,64
**TRY6**	100	69,266667	-30,73333333	30,73
	25	18,300000	-6,7	26,80
	6,25	4,676667	-1,573333333	25,17
	1,52	1,196667	-0,323333333	21,27
	0,39	0,311667	-0,078333333	20,09
	0,098	0,079333	-0,018666667	**19,05**
	0,024	0,023567	-0,000433333	**1,81**
	0,0061	0,006140	4E-05	**0,66**
**RHD**	100	96,20281	-3,79719	**3,80**
	25	25,30366	0,30366	**1,21**
	6,25	6,35008	0,10008	**1,60**
	1,52	1,57688	0,05688	**3,74**
	0,39	0,38539	-0,00461	**1,18**
	0,098	0,10225	0,00425	**4,34**
	0,024	0,0203	-0,0037	**15,42**
	0,0061	0,00599	-0,00011	**1,77**
**LEC3C**	100	103,0088496	3,008849558	**3,01**
	25	23,46394984	1,536050157	**6,14**
	6,25	5,883280757	0,366719243	**5,87**
	1,52	1,462336491	0,057663509	**3,79**
	0,39	0,384440843	0,005559157	**1,43**
	0,098	0,093989983	0,004010017	**4,09**
	0,024	0,021843393	0,002156607	**8,99**
	0,0061	0,005559265	0,000540735	**8,86**
**GSTM1**	100	100,7117438	0,711743772	**0,71**
	25	29,10536779	4,105367793	**16,42**
	6,25	7,789473684	1,539473684	24,63
	1,52	1,967948718	0,447948718	29,47
	0,39	0,487420043	0,097420043	24,98
	0,098	0,130921053	0,032921053	33,59
	0,024	0,028336933	0,004336933	**18,07**
	0,0061	0,005781316	-0,000318684	**5,22**
**GSTT1**	100	99,64157706	-0,358422939	**0,36**
	25	25,1048951	0,104895105	**0,42**
	6,25	6,52173913	0,27173913	**4,35**
	1,52	1,669595782	0,149595782	**9,84**

	0,39	0,413414634	0,023414634	**6,00**
	0,098	0,105851064	0,007851064	**8,01**
	0,024	0,029472759	0,005472759	22,80
	0,0061	0,004835664	-0,001264336	20,73

Supplementary Table S3. Accuracy

It remained below 20% for RHD, and LEC3C. For GSTT1, the difference was below 20% down to 0.098% of input donor DNA and increased but remained below 23% for the two smallest concentrations. GSTM1 and TRY6 showed differences above 20% in the high percentages of input donor DNA but remained under 20% in the small microchimerism percentages, which are the percentages that we expect in our patients. The gene with the worst accuracy results was SRY, as most of the differences calculated were above 20%.

SUPPLEMENTARY FIGURE

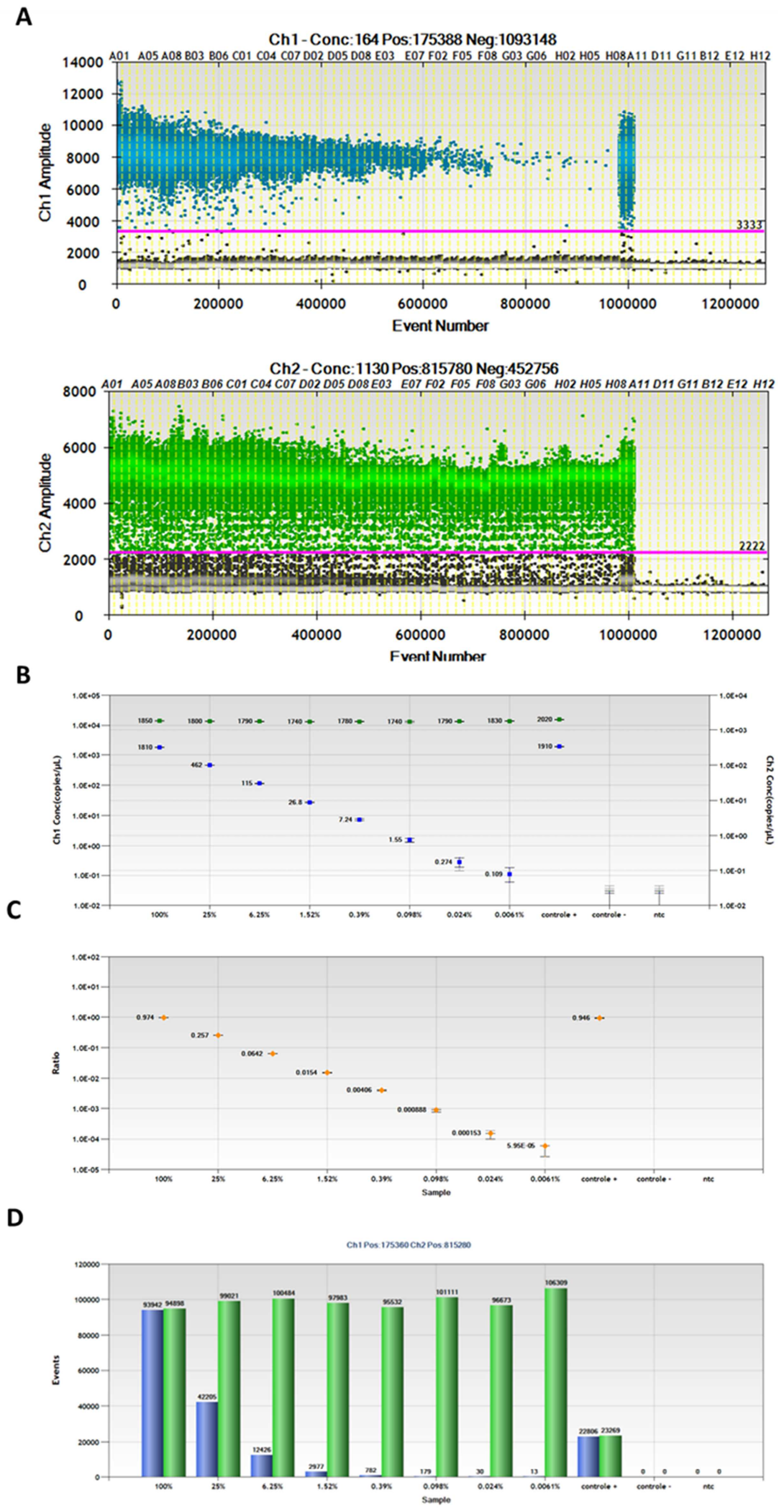

Figure S1: Representative images of the ddPCR results obtained with the Quantasoft software. A) Representation of the fluorescence intensity of each droplet in the sample**.** The positive droplets for the gene of interest (channel 1 : FAM, in blue) show a decrease in

amplitude with dilution, while positive droplets for RNAse P (channel 2 : in green) show a constant amplitude. Grey dots are negative droplets. The pink line shows the positivity threshold. **B)** Concentration of the gene of interest (left y axis) and RNAse P (Right y axis) are plotted as copies / µl **C)** Ratio of the gene of interest over RNAse P **D)** Total number of positive events for the gene of interest and RNAse P.

REFERENCES

1. Hospodsky D, Yamamoto N, Peccia J: **Accuracy, precision, and method detection limits of quantitative PCR for airborne bacteria and fungi.** *Appl Environ Microbiol* 2010, **76:**7004-7012.
